# Contrasting genetic regulation of plant development in wild barley grown in two European environments revealed by nested association mapping

**DOI:** 10.1093/jxb/ery002

**Published:** 2018-01-18

**Authors:** Paul Herzig, Andreas Maurer, Vera Draba, Rajiv Sharma, Fulvia Draicchio, Hazel Bull, Linda Milne, William T B Thomas, Andrew J Flavell, Klaus Pillen

**Affiliations:** 1 Institute of Agricultural and Nutritional Sciences, Martin Luther University Halle-Wittenberg, Halle, Germany; 2 Interdisciplinary Center of Crop Plant Research (IZN), Halle, Germany; 3 The James Hutton Institute (JHI), Invergowrie, Dundee, Scotland, UK; 4 Division of Plant Sciences, University of Dundee at JHI, Invergowrie, Dundee, Scotland, UK

**Keywords:** Cultivated barley (*Hordeum vulgare* ssp. *vulgare*), wild barley (*Hordeum vulgare* ssp. *Spontaneum*), nested association mapping (NAM), plant development, flowering, genome-wide association study (GWAS), quantitative trait locus (QTL), genotype by environment interaction (GxE), genotype by donor interaction (GxD)

## Abstract

Barley is cultivated more widely than the other major world crops because it adapts well to environmental constraints, such as drought, heat, and day length. To better understand the genetic control of local adaptation in barley, we studied development in the nested association mapping population HEB-25, derived from crossing 25 wild barley accessions with the cultivar ‘Barke’. HEB-25 was cultivated in replicated field trials in Dundee (Scotland) and Halle (Germany), differing in regard to day length, precipitation, and temperature. Applying a genome-wide association study, we located 60 and 66 quantitative trait locus (QTL) regions regulating eight plant development traits in Dundee and Halle, respectively. A number of QTLs could be explained by known major genes such as *PHOTOPERIOD 1* (*Ppd-H1*) and *FLOWERING LOCUS T* (*HvFT-1*) that regulate plant development. In addition, we observed that developmental traits in HEB-25 were partly controlled via genotype × environment and genotype × donor interactions, defined as location-specific and family-specific QTL effects. Our findings indicate that QTL alleles are available in the wild barley gene pool that show contrasting effects on plant development, which may be deployed to improve adaptation of cultivated barley to future environmental changes.

## Teaser

Plant development in a barley nested association mapping population is controlled by interacting genotypic (wild donor alleles) and environmental (geographical location) effects.

## Introduction

Barley (*Hordeum vulgare*), a model species for temperate cereals, is an important crop in marginal environments (Baum *et al.*, 2007; Rollins *et al.*, 2013), which are characterised by abiotic stresses such as heat, drought, and nutrient deficiency. These poor and stress-prone environments offer the biggest opportunities for substantially increased yields on a global scale (Tester and Langridge, 2010). In addition, in temperate climates most climate-change scenarios predict increasing average temperatures and elevated risks for extreme weather conditions such as drought and heat (Settele *et al.*, 2014). These ecosystem changes may also affect current high-yielding environments. In comparison with other cereals, barley has a higher adaptability to drought and heat because its early development and extensive root system permit drought escape (Fischer and Maurer, 1978; Lopez-Castaneda and Richards, 1994). Despite the relatively high stress tolerance of barley there is still room for it to be increased further, for instance through fine-tuning of plant development.

The basic reproductive life-cycle of annual plants such as barley can be summarised as germination, vegetative growth, flowering, and seed maturation. In the life-cycle of a plant, flowering time, i.e. the switch between vegetative and reproductive growth, is a key component to adapt and respond to environmental conditions and to optimise the source–sink balance (Cockram *et al.*, 2007), and it is closely linked to agronomic performance and yield (Cuesta-Marcos *et al.*, 2009). The flowering behaviour of barley depends on temperature and photoperiod. Based on photoperiod, barley varieties can be classified into photoperiod-sensitive (early flowering under long-day conditions) and photoperiod-insensitive (day-length neutral, late flowering). Based on vernalisation response, barley varieties can also be classified into winter (vernalisation requirement) and spring (no vernalisation requirement) growth-habit types. In addition, facultative barley varieties exist that exhibit a reduced (or null) vernalisation requirement, which is associated with an acceleration of flowering (Hemming *et al.*, 2009), and generally combined with frost hardiness. The genetic control of the flowering pathway has been extensively studied by mutation studies, quantitative trait locus (QTL) studies, and by exploiting natural genetic variation, resulting in the identification of a number of major genes controlling flowering time (Karsai *et al.*, 2005; Campoli *et al.*, 2012; Comadran *et al.*, 2012; Faure *et al.*, 2012).

In barley, the response to photoperiod under long-day conditions is primarily controlled by the *Photoperiod 1* (*Ppd-H1*) gene. The photoperiod-sensitive and dominant *Ppd-H1* allele promotes flowering (Turner *et al.*, 2005) whereas the photoperiod-insensitive and recessive *ppd-H1* allele delays flowering and maturity, enabling an extension of the growing season towards cooler climates (Jones *et al.*, 2008). The delaying effect of the *ppd-H1* allele is transmitted through a reduction in the expression of *FLOWERING LOCUS T* (*HvFT1*) (Turner *et al.*, 2005). *Ppd-H1* is repressed by circadian clock genes such as *HvELF3*, *HvLUX*, and *PHYTOCHROME C* during the night, and a dysfunctional mutation of a repressor may cause a day-neutral phenotype (Faure *et al.*, 2012; Campoli *et al.*, 2013; Pankin *et al.*, 2014). The circadian clock represents an internal timekeeper that synchronises physiological and molecular processes with the diurnal cycle (Johansson and Staiger, 2015). Under short-day conditions the *Ppd-H2* gene affects flowering (Kikuchi *et al.*, 2009).

The response to cold temperatures is mainly controlled by interaction of the two vernalisation loci in barley, *Vrn-H1* and *Vrn-H2*. *Vrn-H2* is a strong inhibitor of flowering under long-day conditions, functioning as an antagonist of *Ppd-H1* to prevent flowering during cold and harsh conditions (Yan *et al.*, 2004). The expression of the *MADS*-box gene *Vrn-H1* is only induced after extended periods of cold exposure (Oliver *et al.*, 2013), resulting in down-regulation of the flowering repressor *Vrn-H2* (Chen and Dubcovsky, 2012).

The photoperiod and vernalisation pathways converge in the expression of *Vrn-H3*, an orthologue of *HvFT1*, the key integrator of transition from the vegetative to the reproductive phase (Yan *et al.*, 2006). In Arabidopsis, the FT protein moves from leaves to the shoot apical meristem and induces the development of flower primordia. It has been shown that this process is conserved across monocots and dicots (Tamaki *et al.*, 2007; Turck *et al.*, 2008).

In addition, there is a further, temperature-dependent growth regulation pathway active, which is more relevant for short-term ambient temperature changes (Wigge, 2013). This thermo-sensory regulation is poorly understood in barley. However, in Arabidopsis it has been shown that exposure to elevated temperatures may induce early *FT* expression and, subsequently, early flowering independently from day length (Balasubramanian *et al.*, 2006). In this process, increasing temperatures inhibit the ‘evening complex’ of the circadian clock, consisting of *ELF3*, *ELF4*, and LUX (Thines and Harmon, 2010; Herrero *et al.*, 2012). In this way, the expression of *PHYTOCHROME-INTERACTING FACTOR4* (*PIF4*) is induced, which promotes *FT* expression (Box *et al.*, 2015). To date, the integration of thermal signals in the flowering pathway is not fully understood in cereal crops. However, Hemming *et al.* (2012) determined that a *HvFT1* independent signalling pathway exists in barley.

The perception of external signals is important in adapting to environmental conditions and in synchronising optimal plant development. This phenological adaptation, which is required for ‘climate-smart’ agriculture (Olesen *et al.*, 2011), occurs on a genetic level (Zhao and Xu, 2012; El-Soda *et al.*, 2014). In barley, extensive and genetically diverse collections of landraces and wild barley accessions are available (Newton *et al.*, 2011; Russell *et al.*, 2011) that could be used to improve the adaptation potential of cultivated barley and to counteract the reduction of genetic diversity in modern varieties that has resulted from continued selection for favourable agronomic traits.

Barley has become a model for understanding the potential of exotic germplasm to further improve modern elite crops (Ellis *et al.*, 2000; Dawson *et al.*, 2015). Nested association mapping (NAM) is one strategy that places exotic alleles into an elite genetic background in order to investigate their potentially valuable effects in improving complex agronomic traits (Yu *et al.*, 2008; Buckler *et al.*, 2009; Jordan *et al.*, 2011; Nice *et al.*, 2016). The wild barley NAM population HEB-25 was developed for this purpose (Maurer *et al.*, 2015). HEB-25 consists of 1420 BC_1_S_3_ barley lines, comprised of 25 families, each derived from crosses between the cultivar ‘Barke’ (*H. vulgare* ssp. *vulgare*) and one of 25 diverse wild barley (*H. vulgare* ssp*. spontaneum*, *Hsp*) genotypes. A number of studies have been conducted with HEB-25 that confirm the power of the NAM design to detect barley QTLs controlling plant developmental and agronomic traits and to select favourable exotic alleles for subsequent utilisation in barley breeding programs. In this way, favourable exotic alleles have been identified that, for example, improve leaf-rust resistance, flowering time, grain size, and salt-stress tolerance (Schnaithmann *et al.*, 2014; Maurer *et al.*, 2016, 2017; Saade *et al.*, 2016).

The aim of the present study was to investigate plant development in HEB-25 on a location-specific and a family-specific basis and thus to examine the potential ability of the 25 wild barley donors of HEB-25 to improve adaptation to future environmental changes. In this regard, field trials were conducted at two locations, Halle (Germany) and Dundee (Scotland), which differ by 5° in latitude. Latitude is an important environmental driver in determining growth and development (Craufurd and Wheeler, 2009), as it is associated with differences in ambient temperature, photoperiod, and radiation intensity. Additionally, we aimed to study the function of plant developmental genes, segregating in HEB-25, and their impact on plant architecture traits such as height and lodging under diverse environmental conditions.

## Materials and methods

### Plant material

The NAM population ‘Halle Exotic Barley’ (HEB-25; Maurer *et al.*, 2015) was developed by crossing the German spring barley elite cultivar ‘Barke’ (*Hv*) with 25 highly diverse exotic wild barley accessions, namely 24 *Hsp* accessions from the ‘Fertile Crescent’ and Afghanistan and one *H. vulgare* ssp. *agriocrithon* (*Hag*) accession from Tibet (China). After backcrossing the resulting F_1_s with Barke, three consecutive rounds of selfing were conducted by means of single-seed descent. The resulting BC_1_S_3_ plants were propagated as individual bulks to produce 1420 lines, sub-divided into 25 HEB families of 22 to 75 lines. For detailed information about population development, see Maurer *et al.* (2015). In this study, the bulk propagated progeny of BC_1_S_3_ had already undergone several extra generations of selfing and the BC_1_S_3:7_ generation was used for the 2014 experiments. Bulk BC_1_S_3:8_ seed was then used to sow the 2015 experiments.

### HEB-25 field trials

In 2014 and 2015 two field trials in Halle, Germany, and Dundee, Scotland, were carried out under two nitrogen (N) fertilisation treatments to gather phenotypic data. In Halle, the field trial was conducted in randomised complete blocks of 1420 HEB lines for each N treatment. Plots consisted of two rows of 50 seeds with a row length of 1.4 m and a spacing of 0.2 m between rows and 0.5 m between plots. In Dundee, 1371 HEB lines were grown following the same experimental set-up as in Halle. In Dundee, plots consisted of two rows, 40 seeds each, 2.0 m in length, and a distance of 0.25 m between rows and 0.75 m between plots. All field trials were sown in spring, i.e. the end of March in Halle and mid-April in Dundee. Field and disease management were in accordance with local practice.

### Nitrogen treatment

At both locations, HEB-25 was cultivated under two N fertilisation treatments, namely without fertiliser (N0) and with fertiliser (N1). The targeted available nitrogen in N1 was set to 100 kg N ha^–1^. The amount of added fertiliser was calculated after measuring plant available nitrogen in a soil bulk sample before sowing. In Halle, this resulted in fertilising the N1 block with 60kg N ha^–1^ in 2014 and 70 kg N ha^–1^ in 2015. Calcium ammonium nitrate was applied as inorganic N fertiliser at the beginning of the shooting stage. In Dundee, the N1 block was fertilised in 2014 and 2015 with 60 kg N ha^–1^, applied as a 22:4:14 NPK compound mineral fertiliser at sowing. The estimated soil nitrogen content one month before sowing was 34 kg ha^–1^ in 2014 and 62 kg ha^–1^ in 2015, resulting in total available nitrogen in the order of 94 kg ha^–1^ in 2014 and 122 kg ha^–1^ in 2015.

### Environments

The field trials in Halle were conducted at the ‘Kühnfeld Experimental Station’ of the Martin Luther University Halle-Wittenberg (51°29′46.47′′N; 11°59′41.81′′E). In 2014 and 2015 the moderate-to-continental atmospheric growing conditions were characterised by low average temperatures of 6 °C at the beginning of the vegetation period in March and high average temperatures of up to 21 °C in July and August. Almost 50% of the annual precipitation of 514 mm fell during the months July and August (see Supplementary Fig. S1 at *JXB* online). The maximum day length was reached on June 20th with 16.63 h from sunrise to sunset.

In Dundee, the trials were conducted at the Experimental Station of the James Hutton Institute (56°28′53.71′′N; 3°6′35.17′′W). Due to its coastal location, the atmospheric conditions during the trial period can be described as maritime. With regard to the growing season, on average July was the warmest month (16 °C average temperature) and April the coldest (9 °C average maximum). The annual precipitation of 856 and 839 mm in 2014 and 2015, respectively, was equally distributed throughout the year with no discernible maximum. Both years were greater than the 30-year long-term average of 665 mm. The maximum day length was reached on June 20th with 17.45 h of daylight (see Supplementary Fig. S1).

### Phenotyping

Phenotyping of eight agronomic and plant developmental traits was carried out under both treatments as described in Table 1. In Dundee, no lodging was observed and in 2014 the time of shooting was not recorded. Data for maturity in Dundee 2014 were transformed from an ordinal scale into days after sowing. Raw data are archived in the public data repositories e!DAL (Herzig *et al.*, 2017).

**Table 1.** List of eight studied traits and their abbreviations

Trait	Abbreviation	Units	Description	Years/sites studied ^a^	
				2014	2015
Time to shooting	SHO	days	Number of days from sowing until first node noticeable 1 cm above soil surface for 50% of all plants of a plot, BBCH 31 (Lancashire *et al.*, 1991)	H	H, D
Shoot elongation phase	SEL	days	Time from SHO to HEA	H	H, D
Time to heading	HEA	days	Number of days from sowing until awn emergence for 50% of all plants of a plot, BBCH 49 (Lancashire *et al.*, 1991)	H, D	H, D
Ripening phase	RIP	days	Time from HEA to MAT	H, D	H, D
Time to maturity	MAT	days	Number of days from sowing until hard dough: grain content firm and fingernail impression held, BBCH 87 (Lancashire *et al.*, 1991)	H, D	H, D
Plant height	HEI	cm	Average plant height of all plants of a plot measured from soil surface to tip of the erected ear without awns at maturity	H, D	H, D
Lodging	LOD	ordinal	Visual score of lodging as a mean of a plot shortly before harvest (1=no lodging to 9= entire plot lodged)	H	H
Tiller thickness	TCK	ordinal	Visually scored as mean thickness of a bundle of straw harvested from each plot at maturity (1=thin to 9=thick)	–	D

^a^ Location: H, Halle; D, Dundee.

### Statistical analyses

Heritability analyses were performed with SAS 9.4 (SAS Institute Inc., Cary, NC, USA). For each location, broad-sense heritabilities across years were calculated within treatments as:

$$h_{\left(\text{within N}0\text{ or N}1\right)}^{2}=\frac{V_{\text{G}}}{V_{\text{G}}+\frac{V_{\text{R}}}{y}}.$$

and for each location, broad-sense heritabilities across both N levels were calculated as:

$$h_{\left(\text{across N}0\text{ and N}1\right)}^{2}=\frac{V_{\text{G}}}{V_{\text{G}}+\frac{V_{\text{GY}}}{y}+\frac{V_{\text{GT}}}{t}+\frac{V_{\text{R}}}{yt}}$$

where *t* and *y* are the number of N treatments (*t*=2) and years (*y*=2). *V*_G_, *V*_GY_, *V*_GT_, and *V*_R_ correspond to the genotype, genotype × year, genotype × treatment, and error variance components, respectively. All effects were assumed to be random when estimating the variance components using the VARCOMP procedure.

Best linear unbiased estimates (BLUEs) for each HEB line were computed across years for each N level and location and across N levels separately by using the MIXED procedure assuming fixed genotype effects. BLUEs were used for calculation of Pearson’s correlation coefficients (*r*) between traits across N levels, using the R 3.2.4 (www.r-project.org) software with the CORRGRAM package (Friendly, 2002). We also conducted an ANOVA within each location including fixed main effects (year, treatment, and genotype) and all possible single interactions to check for significant genotype × treatment (G×T) effects for each trait.

### Genotyping of HEB-25

Genotyping of 1420 BC_1_S_3_ HEB-25 lines and their 26 parents was conducted by TraitGenetics, Gatersleben, Germany, with the Illumina Infinium iSelect HD 9k chip consisting of 7864 single-nucleotide polymorphism (SNPs; Comadran *et al.*, 2012). After quality checks, 5398 informative SNPs remained for further analyses, as described in Maurer *et al.* (2015). The SNP scores were translated into a quantitative genotype matrix where the state of the homozygous Barke allele was coded as 0, while HEB lines showing a homozygous wild barley genotype were assigned a value of 2. Consequently, heterozygous HEB lines were assigned a value of 1.

### Genome-wide association study (GWAS) analysis

For GWAS, the multiple linear regression Model-A was applied following Liu *et al.* (2011) and Maurer *et al.* (2017). Associations between molecular markers and quantitative traits were determined by *Y* = µ + ∑*X*_*c*_*b*_*c*_ + ε, where *Y* is the phenotypic means vector (i.e. BLUEs), µ is the intercept (equivalent to the overall mean), *X*_*c*_ is the vector containing quantitative marker scores for each individual HEB line at marker *c*, *b*_*c*_ is the allele substitution effect (α) at marker *c*, and ε is the vector of residuals. The GWAS analysis was performed by applying forward–backward regression investigating all SNPs simultaneously, implemented in PROC GLMSELECT in SAS 9.4. SNPs were determined to enter or leave the model if the Schwarz Bayesian Criterion (Schwarz, 1978) was reduced. All SNPs included in the final model were determined as significant. The SNP effect is defined as 2α, where two Barke alleles (genotype 0) are substituted against two exotic alleles (genotype 2) at the respective SNP in the final model. Note that all significant SNP effect estimates are modelled at the same time in the final model. To estimate the explained phenotypic variance (*R*^2^) for each marker–trait association, the significant SNP was modelled solely in a linear model.

To assess the accuracy of the prediction of all traits by GWAS, a cross-validation (CV) approach was applied following Maurer *et al.* (2017). A five-fold cross-validation was conducted 20 times (100 CV runs) where 80% of the HEB lines, randomly selected within each family, formed the estimation set (ES). This ES was used for detection of QTLs and effect estimation. The remaining 20% of the lines represented the prediction set (PS), wherein the phenotype was predicted based on the SNP effects estimated in the ES. The explained phenotypic variance (*R*^2^_adj_) was determined as the modelled fit of the predicted phenotypic value of all significant markers simultaneously regressed on the observed phenotypic value within each ES. Prediction ability (*R*^2^_pred_) was evaluated by predicting the phenotype of the PS by means of detected SNP effects from the ES. *R*^2^_adj_ and *R*^2^_pred_ were averaged across all 100 CV runs to calculate the final values. The number of significances for each marker was counted across all CV runs and was defined as the detection rate (DR). The presence of a reliable QTL was assumed next to a peak marker with a DR of ≥25. Single-marker *P*-values, *R*^2^, and effect estimates were taken across all runs where the respective marker was significant. To identify potential candidate genes we used BARLEYMAP (Cantalapiedra *et al.*, 2015).

### Calculation of family-specific donor allele QTL effects

As a consequence of the NAM population design, we expected a variation of allelic QTL effects among the 25 wild donor-derived NAM families. Therefore, we cumulated significant SNP marker effects and estimated family-specific donor allele QTL effects following the approach of Maurer *et al.* (2017). The cumulating procedure started with the identification of the peak marker, which is defined as the SNP with the highest DR across all CV runs. This peak marker formed the centre of a 26-cM interval, which was scanned for further significant SNPs. All Model-A SNP effect estimates within this interval were then cumulated for each of the 25 donors separately, as ∑^*n*^_*i*_ SNP(donor)_*i*_ × α_*i*_, where *i* iterates through all significant SNPs (*n*) in the same interval, SNP(donor)_*i*_ represents the donor genotype (0 or 2) of the *i*-th significant SNP, and α_*i*_ is the SNP effect, obtained from Model-A. This method is based on the fact that different donors show different segregation patterns across significant SNPs, ultimately leading to different donor-specific effect estimates for each QTL. The family-wise cumulating was done for each CV run and the mean of them was taken as the final family-specific QTL effect estimate. To test whether family-specific QTL effects were significantly different between locations a simple paired *t*-test was performed.

### Grouping different QTLs into QTL regions

Peak markers significant for different traits and located within an interval ≤10 cM were combined to one QTL region, which was considered to regulate multiple traits simultaneously.

## Results and discussion

### Descriptive statistics

The HEB-25 population was cultivated under two contrasting nitrogen treatments, in both Halle and Dundee. Overall, we observed a wide phenotypic variation for all studied traits throughout the trials (see Supplementary Fig. S2), which resulted in relatively high coefficients of variation (CoV) that were >10% for the traits SEL, HEI, LOD, and SHO in Dundee (Table 2; see Table 1 for abbreviations). In contrast, MAT revealed the lowest CoV (<4.5%). Significant (*P*<0.05) genotype effects were observed for all traits in both locations. Likewise, treatment effects were significant for all traits in both locations, except for SEL in Halle (Supplementary Table S1). In contrast, significant genotype × treatment (G×T) interactions were only observed for HEA and MAT in both locations and for RIP in Halle (Supplementary Table S1). The marginal difference between nitrogen treatments for all developmental stages may indicate that nitrogen fertilisation at this magnitude exhibited only minor effects on plant development, which is also confirmed and summarised in Hall *et al.* (2014). In contrast to common literature (Takeno, 2016) and our expectations, non-fertilised plants were, on average, not affected in plant development, indicating that no severe nitrogen stress was induced in our trials. In addition, the lack of strong G×T interactions in Dundee and in Halle indicated that the HEB lines generally reacted similarly to the increase of N supply from the N0 to the N1 treatment. For simplicity of data interpretation, we thus based the following GWAS analysis on phenotype data averaged across treatments (BLUEs were used), excluding SNP × treatment effects.

**Table 2.** Descriptive statistics for the eight traits studied, including best linear unbiased estimates (BLUEs) and heritability

Trait ^a^	Location	N level	*N*	Mean	SD	Min	Max	CoV ^b^	h^2 c^	G×T ^d^
SHO	Halle	N0	1420	56.6	5.2	42	74	9.2	81.9	n.s.
		N1	1420	54.8	5.1	43	74	9.3	83.7	
		Across	1420	55.6	5.0	43	74	9.0	88.2	
	Dundee	N0	1371	50.4	6.2	31	70	12.4	–	n.s.
		N1	1371	51.5	6.3	31	70	12.3	–	
		Across	1371	50.9	5.2	31	70	10.2	–	
SEL	Halle	N0	1420	14.1	2.9	5	24	20.8	59.2	n.s.
		N1	1420	14.1	2.9	5	22	20.8	52.1	
		Across	1420	14.0	2.6	7	23	19.0	67.2	
	Dundee	N0	1371	36.2	5.5	19	53	15.2	–	n.s.
		N1	1371	34.8	5.8	17	53	16.7	–	
		Across	1371	35.5	4.5	23	53	12.7	–	
HEA	Halle	N0	1420	70.5	5.3	56	85	7.5	91.0	**
		N1	1420	68.6	5.2	52	85	7.6	90.7	
		Across	1420	69.4	5.2	54	84	7.5	93.1	
	Dundee	N0	1371	79.0	4.8	65	94	6.1	83.3	***
		N1	1371	77.8	4.5	65	92	5.8	86.8	
		Across	1371	78.4	4.5	65	93	5.8	89.2	
RIP	Halle	N0	1420	37.6	2.8	28	49	7.5	42.5	***
		N1	1420	37.3	2.9	25	47	7.6	51.2	
		Across	1420	37.3	2.6	30	46	6.8	57.2	
	Dundee	N0	1371	50.1	4.9	33	68	9.8	36.6	n.s.
		N1	1371	46.4	5.0	32	66	10.7	28.8	
		Across	1371	48.3	4.1	36	66	8.5	46.3	
MAT	Halle	N0	1420	107.9	4.6	95	121	4.3	73.3	***
		N1	1420	105.8	4.1	94	118	3.9	76.7	
		Across	1420	106.7	4.2	95	119	4.0	81.7	
	Dundee	N0	1371	129.2	4.8	111	146	3.7	23.7	***
		N1	1371	124.1	5.1	109	142	4.1	25.2	
		Across	1371	126.6	4.1	113	141	3.2	38.4	
HEI	Halle	N0	1420	70.0	11.1	44	110	15.9	82.2	n.s.
		N1	1420	70.6	10.8	38	108	15.2	79.9	
		Across	1420	70.2	10.6	42	106	15.1	86.2	
	Dundee	N0	1371	69.8	12.5	38	119	18.0	74.8	n.s.
		N1	1371	81.3	13.1	50	124	16.1	75.1	
		Across	1371	75.5	12.0	51	114	15.9	84.9	
LOD	Halle	N0	1420	2.6	0.9	1	6	33.6	58.1	n.s.
		N1	1420	2.7	0.9	1	7	33.2	59.5	
		Across	1420	2.6	0.8	1	6	30.4	71.1	
TCK	Dundee	N0	1371	5.5	2.2	1	9	39.5	n/a	n/a

^a^ Trait abbreviations are given in Table 1. ^b^ Coefficient of variation in %. ^c^ Broad-sense heritability in %. ^d^ Significant Genotype × Treatment interactions: *P<0.05; **P<0.01; ***P<0.001; n.s., not significant.

Differences in plant development were observed between locations. During the early stages of the growing period, HEB lines developed faster in Dundee and reached SHO on average 4.7 d earlier than in Halle (Table 2). Later on, this trend was reversed, with plants in Dundee remaining in the SEL phase on average 21.5 d longer than in Halle, so HEA occurred on average 9.0 d later than in Halle. Corresponding delays to maturation were also seen for Dundee (average +19.9 d relative to Halle). One explanation for different plant development in Halle and Dundee may be the variation of photoperiod between the two locations (Villegas *et al.*, 2016). In total, 355 (25%) out of 1420 HEB lines carry a photoperiod-sensitive allele of *Ppd-H1* (Maurer *et al.*, 2015), the main driver of photoperiod response under long-day conditions in barley (Turner *et al.*, 2005). In Halle, long-day conditions (>12 h light) prevailed during sowing, with a progressive increase in day length. In Dundee, day length had already reached 13.5 h at the time of sowing and the daily increase was more pronounced than in Halle (see Supplementary Fig. S3). This might explain the faster plant development until the SHO stage in Dundee. However, it is unlikely that only 25% of the population carrying the photoperiod-sensitive exotic allele of *Ppd-H1* could cause an average location difference of 4.7 d across all 1420 HEB lines. Therefore, we assume that other abiotic factors such as soil temperature, ambient temperature, and precipitation may have also contributed to the generally accelerated plant development in Dundee. For example, Supplementary Fig. S1 shows that the average temperature in Halle was lower in winter than in Dundee, suggesting that the soil temperature was lower and therefore took longer to increase in spring, and hence early growth could have been delayed in Halle compared to Dundee. Hemming *et al.* (2012) observed faster reproductive plant development under elevated temperatures, which might explain the shift in developmental differences between Halle and the comparatively cooler location of Dundee (Supplementary Table S1). Furthermore, in Halle the SEL phase was less than half as long as in Dundee, with only minor differences in HEI between both locations, indicating a slower growth rate in Dundee (Table 2).

In Halle, the occurrence of lodging (LOD) was relatively low, with 2.6 in N0 (low N) and 2.7 in N1 (high N). All traits showed higher broad-sense heritability across than within nitrogen treatments (Table 2). In general, traits showed heritability estimates >50%, except for RIP in Dundee and Halle (N0), and MAT in Dundee. In addition, higher trait heritabilities were observed in Halle compared to Dundee, with the maximum value being observed for HEA across treatments, with *h*^2^=93.1%.

### Trait correlations

Pearson’s correlation coefficients between traits were calculated across treatments for each location separately (Table 3). In addition, the cross-correlation of a trait between locations was determined. In Halle, the highest correlations were observed between SHO and HEA and SHO and MAT, indicating that early-shooting genotypes tended to be early in HEA and MAT and late-shooting genotypes tended to be late in HEA and MAT. Maurer *et al.* (2016) observed similar relationships between developmental traits in Halle. In Dundee, this relationship was diminished with medium correlations between SHO and HEA (*r*=0.61) and HEA and MAT (*r*=0.54) and a low correlation between SHO and MAT (*r*=0.31). The traits RIP and SEL were calculated as the differences between two other stages of development, and hence correlations can provide some indications for their main determinant. For example, in Halle RIP was predominantly determined by HEA (–0.59), and SEL was equally determined by SHO and HEA. In contrast, in Dundee RIP had a medium correlation with both determinants, and HEA, MAT, and SEL were mainly determined by SHO. This suggests that MAT was strongly influenced by environmental factors such as heat and drought in Halle, as already mentioned in Maurer *et al.* (2016). In Dundee, equally distributed precipitation throughout the year and lower temperatures did not induce heat or drought stress during the maturity phase, so the vegetation period in total lasted 20 d longer than in Halle. Thus, in Dundee MAT might have been under stronger control of genetic factors than in Halle. The difference in plant development between the two locations was further supported by the fact that SEL and HEI showed a positive correlation (*r*=0.50) in Halle, but no detectable correlation (*r*=–0.08) in Dundee. This suggests that in Dundee plant growth was maintained after flowering, while in Halle it was essentially terminated during flowering. Moreover, as expected, LOD and HEI showed a relatively high correlation (*r*=0.65) in Halle, whereas tiller thickness (TCK) in Dundee was independent from HEI (*r*=0.14).

**Table 3.** Pearson**’**s correlation coefficients (*r*) between eight developmental traits based on trait BLUEs across nitrogen levels. Shaded values are cross-correlations between locations; values below this diagonal (i.e. bottom-left) are calculated separately for Halle and values above the diagonal (i.e. top-right) are calculated separately for Dundee

Trait ^a^	HEI		SHO		HEA		MAT		SEL		RIP		TCK	
HEI	0.83	***	0.03	n.s.	**–0.07**	**	**–0.05**	*	**–0.08**	**	0.02	n.s.	0.14	***
SHO	**–0.29**	***	0.46	***	0.61	***	0.31	***	**–0.48**	***	**–0.32**	***	0.22	***
HEA	**–0.03**	**n.s.**	0.86	***	0.88	***	0.54	***	0.12	***	**–0.54**	***	0.33	***
MAT	**–0.13**	***	0.79	***	0.87	***	0.53	***	0.08	**	0.41	***	0.18	***
SEL	0.50	***	**–0.21**	***	0.29	***	0.17	***	**–0.02**	**n.s.**	**–0.05**	*	**–0.01**	**n.s.**
RIP	0.14	***	**–0.44**	***	**–0.59**	***	**–0.12**	***	**–0.32**	***	0.51	***	**–0.17**	***
LOD	0.65	***	**–0.52**	***	**–0.34**	***	**–0.40**	***	0.31	***	0.03	n.s.	n/a	

^a^ Trait abbreviations are given in Table 1. Significant correlation coefficients are indicated: *P<0.05; **P<0.01; ***P<0.001; n.s., not significant. Negative correlations are highlighted in bold.

When comparing trait performances between Halle and Dundee, we observed strong cross-correlations for HEI (*r*>0.83) and HEA (*r*>0.88), while for SHO, MAT, and RIP medium cross-correlations between *r*=0.46 and *r*=0.53 were observed. This finding indicates that HEI and HEA were controlled largely by environmentally unresponsive QTLs while the remaining traits responded to location-specific QTLs.

### QTL detection through GWAS

**Fig. 1.**
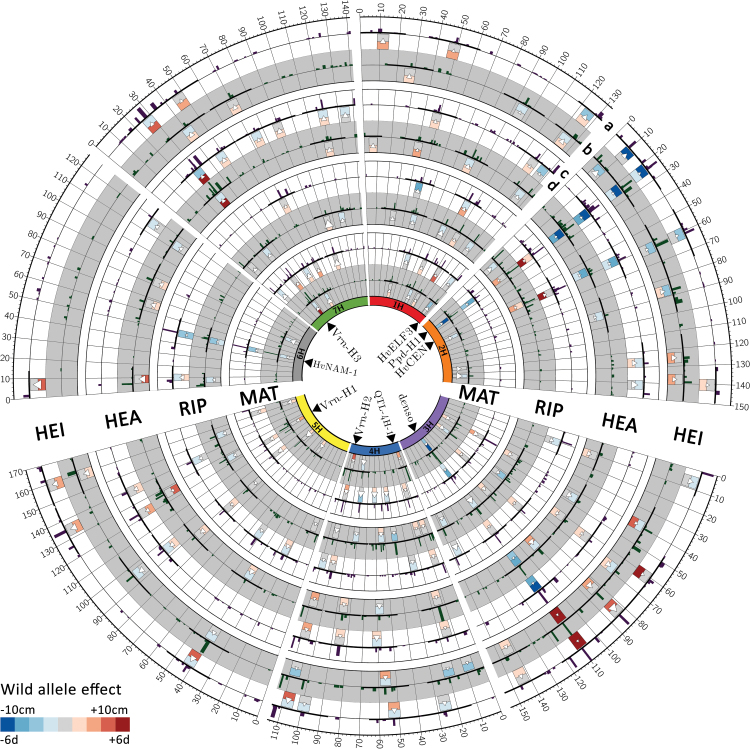
GWAS results of developmental traits and plant height. Candidate genes of major QTLs are indicated in the centre of the circle. Barley chromosomes are shown as coloured bars on the inner circle. Concentric rings in white represent results for Dundee (a) and rings in grey results for Halle (b). Histograms in the outer part of the concentric rings (c) indicate the frequency of QTL detection rates in 100 cross-validation runs during the GWAS procedure: QTLs with a detection rate ≥25 are defined as reliable. Family-specific effects are indicated as colored boxes in the inner part of the concentric rings (d): the minimum and the maximum effects across the 25 families are represented as heat maps in the upper and lower part of the boxes, respectively. The size of the triangles symbolises the coefficient of variation across all family effects.

A GWAS was carried out for all traits to compare the genetic control of plant development between Halle and Dundee. A multiple linear regression model was used, followed by a HEB family-specific estimation of the QTL effect to exploit the full potential of the NAM design. In total, 491 significant marker–trait associations were identified with a detection rate ≥25 (cross-validation) for eight traits, across Dundee and Halle (Supplementary Table S2). Subsequently, these marker–trait associations were converged into 122 and 164 QTLs (for all traits) and placed on 60 and 66 QTL (across traits) regions regulating eight plant development traits in Dundee and Halle, respectively (Table 4, Fig. 1, and Supplementary Tables S3–4). We also re-analysed the HEB-25 phenotype data using growing degree-days (GDD) as outlined by Maurer *et al.* (2016). However, we detected similar differences in growth rates for Halle and Dundee, and the QTLs listed in Table 4 were almost identical using GDD and regular days (data not shown). We thus conclude that the extended growth phase in Dundee was primarily a location-specific effect rather than a thermal effect. The phenotypic variance explained by a full QTL model (*R*^2^_adj_) reached higher percentages in Halle, with a range from 62.6% (RIP) to 82.8% (HEA), than in Dundee, which had a range from 19.6% (SEL) to 81.1% (HEA, Table 4). The calculation of the prediction ability (*R*^2^_pred_) revealed a similar result. In Halle, *R*^2^_pred_ values were relatively high, affirming the robustness of the method (Maurer *et al.*, 2016) and the reliability of the detected QTL results. In Dundee, *R*^2^_pred_ values were low in general, except for HEA and HEI. For SHO, SEL, and TCK the results seemed to be less reliable. A major cause of the decreased explained phenotypic variances of the latter traits was probably the phenotypic data, which were based on only one year in Dundee. In addition, mathematically derived traits in general have less power in QTL studies (Wang *et al.*, 2011), potentially reducing the explained phenotypic variances of SEL and RIP.

**Table 4.** Number of QTLs, explained phenotypic variance, and prediction ability per trait and location

Trait ^a^	Location	Analysis type ^b^	QTLs ^c^	*R* ^2^_adj_^d^	*R* ^2^_pred_^e^
SHO	Halle	Across	28	81.3	63.3
	Dundee	Across	12	41.8	18.7
SEL	Halle	Across	23	70.9	45.9
	Dundee	Across	11	19.6	0.4
HEA	Halle	Across	25	82.8	65.5
	Dundee	Across	23	81.1	63.4
RIP	Halle	Across	29	62.6	33.6
	Dundee	Across	19	52.4	26.8
MAT	Halle	Across	22	75.9	56.5
	Dundee	Across	22	53.6	23.1
HEI	Halle	Across	23	82.4	67.0
	Dundee	Across	22	79.7	64.8
LOD	Halle	Across	16	70.9	52.0
TCK	Dundee	N0	15	44.5	17.5

^a^ Trait abbreviations are given in Table 1. ^b^ Phenotypic data used for GWAS were analysed across N treatments or restricted to N0 (low N). ^c^ Number of robust QTLs with detection rate of peak marker ≥25. ^d^ Mean explained phenotypic variance in the training set across all cross-validation runs in %. ^e^ Mean prediction ability in the validation set across all cross-validation runs in %.

Among the traits, we observed both decreasing and increasing *Hsp* (wild barley) allele QTL effects compared to the elite Barke allele ( Supplementary Tables S3–4). Most of the major QTL regions showed a consistent, location-independent effect for all developmental traits, with a low CoV across families. This was true, for instance, for *Ppd-H1*, *sdw1*, and *HvCEN*, which were originally described by Laurie *et al.* (1995). Furthermore, we obtained a broad range of family-specific QTL effects (Supplementary Tables S3–4). For instance, in Dundee the *Hsp* allele at the *Vrn-H3* locus (QTL-7H-3) increased HEA by 5.4 d in the HEB family 04 but reduced HEA by 2.0 d in family 24. Quite often, QTL regions exerted effects on several traits simultaneously. Therefore, in the following sections we discuss in detail trait-specific QTL effects that were present in selected prominent QTL regions. These regions are summarised in Table 5. Details for all QTLs and QTL regions can be found in Supplementary Tables S3–4 and Fig. S4.

**Table 5.** List of selected QTL regions with mean, minimum, and maximum family-specific effects on eight developmental traits across treatments

QTL region ^ a ^	QTL intervall ^ b ^	Loc ^ c)^	SHO ^ d ^				SEL ^ d ^				HEA ^ d ^				RIP ^ d ^				MAT ^ d ^				HEI ^ d ^				LOD (in HAL) and TCK (in DUN) ^ e ^			
			Ø	Min	Max	CoV	Ø	Min	Max	CoV	Ø	Min	Max	CoV	Ø	Min	Max	CoV	Ø	Min	Max	CoV	Ø	Min	Max	CoV	Ø	Min	Max	CoV
**QTL-2H-2 (*Ppd-H1***)	23.0–23.8	DUN	**–5.8**	**–7.6**	**–3.2**	0.2	**–0.7**	**–2.7**	1.0	1.0	**–7.6**	**–8.7**	**–4.3**	0.1	4.2	1.4	5.1	0.2	**–3.0**	**–3.8**	**–2.1**	0.2	**–6.8**	**–8.3**	**–1.7**	0.2	**–1.4**	**–1.8**	**–0.8**	0.2^TCK^
		HAL	**–6.1**	**–7.1**	**–2.2**	0.2	**–2.1**	**–2.2**	**–0.1**	0.2	**–8.5**	**–9.8**	**–4.0**	0.1	2.4	0.4	2.8	0.2	**–6.3**	**–7.5**	**–2.7**	0.2	**–6.4**	**–8.6**	**–3.1**	0.1				
**QTL-2H-3 (*HvCEN***)	57.0–62.7	DUN	**–1.1**	**–1.8**	**–0.4**	0.3	**–0.8**	**–1.7**	0.5	0.6	**–2.3**	**–3.5**	**–0.8**	0.2	2.1	0.3	6.4	0.5					**–3.0**	**–5.1**	**–0.5**	0.4	**–1.6**	**–1.8**	**–0.7**	0.2 ^TCK^
		HAL	**–0.6**	**–1.6**	0.2	0.8	**–2.1**	**–2.9**	**–1.3**	0.2	**–2.6**	**–3.4**	**–0.8**	0.2	1.5	0.9	2.2	0.2	**–0.8**	**–1.7**	0.0	0.6	**–3.2**	**–4.9**	**–0.9**	0.3				
**QTL-3H-5 (*uzu***)	51.5–58.3	DUN	0.5	**–0.1**	3.6	1.5																					1.3	**0.0**	1.9	0.3 ^TCK^
		HAL					0.2	**–0.1**	1.0	1.4																				
**QTL-3H-11 (*sdw1***)	106.1– 109.2	DUN	**–2.6**	**–3.6**	0.2	0.3	**–0.9**	**–1.6**	0.1	0.5	**–4.3**	**–5.6**	**–3.8**	0.1	1.4	0.6	1.8	0.1	**–2.9**	**–3.6**	**–1.7**	0.1	20.8	15.8	21.9	0.1				
		HAL	**–5.6**	**–6.0**	**–5.0**	0.1	2.5	1.5	2.8	0.1	**–3.4**	**–3.7**	**–2.7**	0.1	**–0.1**	**–1.0**	0.1	1.8	**–3.2**	**–5.6**	**–2.8**	0.2	18.1	13.4	19.0	0.1	1.1	0.9	1.5	0.1 ^LOD^
**QTL-4H-4 (*PhyB***)	50.9–60.8	DUN	0.3	**–4.1**	3.4	1.6					0.0	**–1.5**	0.7	1.6					**–0.1**	**–2.2**	0.3	1.8	0.6	**–1.5**	3.3	1.3	**–0.6**	**–1.3**	0.6	0.6 ^TCK^
		HAL	**–0.1**	**–0.7**	0.3	1.3					0.0	**–1.0**	0.5	1.3	0.6	0.0	1.4	0.6	0.0	**–0.9**	0.6	1.4	1.0	**–1.4**	3.9	0.9				
**QTL-4H-8 (*Vrn-H2***)	111.3– 115.2	DUN	0.9	0.0	2.0	1.0					0.9	**–0.2**	2.3	0.8	**–0.8**	**–1.6**	0.0	0.4					**–0.2**	**–1.6**	6.4	1.7				
		HAL	1.4	0.0	2.7	0.4					1.4	0.0	2.1	0.3									**–3.1**	**–4.5**	0.0	0.4	**–0.2**	**–0.3**	**0.0**	0.3 ^LOD^
**QTL-6H-1**	0.9–8.6	DUN													**–0.4**	**–1.2**	1.5	0.9					0.3	**–0.2**	6.7	4.0				
		HAL	0.2	**–0.2**	3.5	2.7					0.3	**–0.4**	4.3	1.9	**–0.5**	**–0.8**	**–0.1**	0.3												
**QTL-7H-3 (*Vrn-H3***)	27.6–34.3	DUN	1.1	0.6	2.0	0.4	**–0.1**	**–1.7**	2.3	2.1	2.1	**–2.0**	5.4	0.8					1.6	**–0.2**	2.8	0.5	1.9	**–2.3**	5.7	0.9				
		HAL	2.2	**–0.1**	3.7	0.5	0.1	**–0.6**	1.6	1.2	2.3	**–1.5**	6.6	0.8	**–0.6**	**–1.2**	0.1	0.5	1.8	**–1.0**	4.5	0.6								

^a^ QTL region including chromosomal designation and index. ^b^ QTL interval in cM, based on Maurer *et al.* (2015). ^c^ Location: HAL, Halle, DUN, Dundee. ^d^ Ø, Min, Max, and CoV indicate mean, minimum, maximum, and coefficient of variation of the exotic QTL effect allele, defined by substituting the two Barke alleles against the two exotic alleles and calculated across all families. Negative exotic QTL allele effects are indicated in bold. ^e^ QTL regions controlling TCK or LOD are indicated by the abbreviations. Trait abbreviations are given in Table 1.

### QTL region 2H-2 (*Ppd-H1*)

This well-known QTL turned out to be a main driver for developmental traits under long-day conditions (see Supplementary Table S4).All developmental traits studied except LOD were regulated at this QTL. The most significant marker in this QTL region was SNP BK_16, which was directly located within the *Ppd-H1* gene and explained up to 42% of the phenotypic variance for HEA ( Supplementary Table S2). *Ppd-H1* mainly controls sensitivity to long photoperiods (Turner *et al.*, 2005), accelerating reproductive development when subjected to long days. We compared the effect of the *Hsp* allele of *Ppd-H1* on later phases of plant development between locations and observed that, on average, MAT was accelerated by 6.3 d and RIP was extended by 2.4 d in Halle, whereas in Dundee MAT was accelerated by 3.0 d and RIP was extended by 4.2 d. These effects were detectable in all HEB families. The less-pronounced accelerating effect in Dundee deviated from the expectation with regard to photoperiod responses ( Supplementary Fig. S3). During sowing, the day length in Dundee (13.8 h) was longer than in Halle (12.3 h) and the difference slightly increased, reaching a maximum at 75 d. According to the literature, extending the day length leads to a faster life-cycle in photo-sensitive genotypes (Roberts *et al.*, 1988; Paynter *et al.*, 2001). We thus expected a stronger *Hsp* effect in Dundee than in Halle. We conclude that the response of the photoperiod-sensitive exotic *Ppd-H1* allele to an increase in day length in Dundee may have been counteracted by other environmental cues, for instance the effect of ambient temperature on plant development. Halle is characterised by higher temperatures during the growing season than Dundee, in particular during early development (April) and maturity (July) ( Supplementary Fig. S1). Hemming *et al.* (2012) and Ford *et al.* (2016) found out that high temperatures resulted in rapid progression of reproductive development under long-day conditions through a gene × environment interaction. This interaction indicates not only a day length-dependent but also a temperature-dependent reaction of *Ppd-H1*. High temperature could have caused faster development in Halle and counterbalanced the accelerating day-length effect in Dundee. Interestingly, in addition to its strong effects on reproductive development, the *Hsp* alleles of *Ppd-H1* also exerted strong effects on HEI (–6.8 cm) and TCK (–1.4 score). These effects may be attributed to the faster development induced by the *Hsp* alleles of *Ppd-H1*, which accelerates progression through plant development, thereby reducing terminal height and tiller thickness. Furthermore, the exotic donor allele present in HEB family 13 produced the smallest plant development effects across all the HEB donors (Supplementary Table S3). This observation may indicate that the donor *Ppd-H1* allele in HEB family 13 has a higher similarity to the Barke allele. Preliminary exome capture SNP data support this assumption since we found only two SNPs for *Ppd-H1* in HEB family 13 (Supplementary Table S5).

### QTL region 2H-3 (*HvCEN*)

This association was identified as a robust and stable pleiotropic effect controlling all the traits studied except LOD. The direction of *Hsp* effects at QTL-2H-3 were consistent with *Ppd-H1*, although expressed to a lesser extent, except for TCK where the magnitude of effects were similar to *Ppd-H1*. The most likely candidate gene is *HvCEN* (Comadran *et al.*, 2012). The *Hsp* allele seemed to accelerate early reproductive stages, both in Dundee and in Halle, whereas the effect on MAT was very low and only detected in Halle. The difference between locations was largest for SEL and RIP, with *Hsp* reducing SEL and extending RIP in Dundee. Strong family-specific and location-specific effects were observed for RIP. The *Hsp* allele present in HEB family 11 produced a strong effect in Dundee, where RIP was extended by 6.4 d compared to the Barke allele. The effect of the donor allele in HEB family 11 was at least 4.0 d stronger than for all other *Hsp* alleles present in the HEB-25 population. The donor allele is derived from the *Hsp* accession HID109, originating from Syria (Maurer *et al.* (2015). We found a unique *HvCEN* SNP at position 523 378 414 bp, which was only present in HEB family 11 (Supplementary Table S5). This SNP may explain the strong effect on extending the ripening phase in Dundee (Supplementary Table S3). It would be interesting to mine further alleles from Syrian wild barleys, which are potentially useful to adjust the length of the ripening period. In contrast, the *Hsp* allele effect was strongly reduced in Halle, where RIP was only extended by 2.3 d in HEB family 11. It turned out that the extension of RIP was achieved at the expense of SEL. The same *Hsp* allele in family 11 exerted the strongest effects in the HEB-25 population, reducing SEL to 1.7 and 2.9 d in Dundee and Halle, respectively. To conclude, the family-specific and location-specific *Hsp* effect of donor HID109 might be of particular interest for barley breeders to enable a considerable extension of the ripening phase in Northern European environments.

### QTL region 3H-5 (*uzu*)

We observed a QTL region that showed a significant effect on tiller thickness (TCK). Family-specific *Hsp* effects ranged from 0 (HEB family 01) to a score of +1.9 (HEB family 24). Thus, this QTL represents the main positive driver to increase tiller thickness, a trait supportive to increases in stem strength and lodging resistance. The most likely candidate in this region is the *uzu* gene. The *uzu* mutant induces a semi-dwarf, upright plant architecture with an improved canopy structure and sturdy straw due to its brassinosteroid deficiency (Chono *et al.*, 2003; Saisho *et al.*, 2004). The *uzu* gene is mainly observed in six-rowed barley varieties originating from East Asia, including Japan, Korea, and China (Takahashi, 1951; Dockter and Hansson, 2015). Interestingly, the donor of family 24, a *H. vulgare* ssp. *agriocrithon* accession originating from Tibet, showed the strongest positive effect on TCK (Supplementary Table S3).

### QTL region 3H-11 (*sdw1*)

QTL region 3H-11 produced the strongest effect on plant height, with the *Hsp* allele increasing HEI on average by 20.8 cm in Dundee and 18.1 cm in Halle. The most likely candidate gene explaining this effect is the *sdw1* locus (also called *denso*), encoding a GA-20 oxidase (Jia *et al.*, 2015). The semi-dwarf allele, present in the Barke cultivar, contributed to the ‘Green Revolution’ in the 1960s by reducing plant height and, at the same time, increasing lodging resistance, harvest index, and grain yield in modern cultivars (Milach and Federizzi, 2001). Two exotic donor alleles at the *sdw1* locus, present in HEB families 08 and 24, still produced increasing plant height effects in Dundee (+15.82 and +16.37 cm, respectively) and Halle (+13.38 and +13.88 cm, respectively) relative to the Barke *sdw1* allele. However, these effects were much less pronounced than in the remaining HEB families, which had maximum exotic allele effects of +21.91 and +18.99 cm in Dundee and Halle, respectively (see Supplementary Table S3). We found a unique exotic SNP for *sdw1* at position 634 073 570 bp, which was only present in HEB families 08 and 24 (see Supplementary Table S5). This exotic SNP may explain the less-pronounced effect of these alleles in comparison with the remaining HEB donor alleles.

In addition, wild alleles seem to have a positive effect on thousand-grain weight (Maurer *et al.*, 2016). The *Hsp* effects on developmental traits differed clearly between Halle and Dundee. In Dundee, the effect of *Hsp* in reducing SHO (–2.6 d) was less pronounced than in Halle (–5.6 d). SEL was reduced in Dundee (–0.9 d), while it was prolonged in Halle (2.5 d). Finally, RIP was extended by 1.4 d in Dundee, whereas no clear *Hsp* effect was observed in Halle. The *sdw1* locus might be a further example of a day-length × temperature interaction, with changing impacts on different developmental traits. Wang *et al.* (2010) found suggestions for such a relationship by varying sowing time. A further interesting aspect is that HEB family 24, developed from a *H. vulgare* ssp. *agriocrithon* accession, showed less-pronounced effects at QTL region 3H-11 for most of the studied traits. This may indicate the presence of a *sdw1* allele that is more related to Barke at this position than the *Hsp* alleles present in the remaining HEB families.

### QTL region 4H-4

This QTL region showed minor *Hsp* effects on most developmental traits. However, contrasting family-specific *Hsp* effects were obtained in Dundee; for example, *Hsp* effects on SHO ranged from –4.1 d (HEB family 17) to +3.4 d (HEB family 08). This finding indicates that contrasting wild barley donor alleles were present in the HEB-25 population, which may be particularly suited to support contrasting breeding goals – in this case early versus late juvenile development. A couple of candidate genes were located adjacent to the peak marker of QTL region 4H-4 (SNP 11_10261 at 50.85cM). One candidate, *phytochrome B* (*PhyB*), is a red/far-red light receptor, which induces photoperiod-dependent flowering (Szucs *et al.*, 2006; Karsai *et al.*, 2008). The different day lengths and the associated light qualities at Dundee and Halle may cause location-specific effects. *PhyB* also plays a role in the frost-tolerance pathway in barley (Novak *et al.*, 2016). A second candidate, *HvCO16*, belongs to the *CONSTANS*-like (*COL*) gene family. *COL* genes possess CCT domains and are known to promote flowering under long-day conditions in plants (Putterill *et al.*, 1995; Griffiths *et al.*, 2003; Cockram *et al.*, 2012). Two additional candidate genes, *HvPRR59* and *HvPRR73*, belonging to the pseudo-response regulator family (*PRR*s), are also located in QTL region 4H-4. *PRR*s are part of the circadian clock, a pathway active in plants to perceive and respond to day-length and temperature cues (Song *et al.*, 2015). These two candidate genes are Arabidopsis orthologues, indicating that functions of the clock may be conserved (Campoli *et al.*, 2012; Calixto *et al.*, 2015).

### QTL region 4H-8 (*Vrn-H2*)

QTL-4H-8 showed significant effects on HEI, SHO, and HEA at both locations and, in Dundee only, on SEL and RIP. SHO and HEA were delayed by the *Hsp* allele, with a maximum extension of SHO by 2.7 d in Halle in HEB family 23. The QTL effects can presumably be explained by the candidate gene *Vrn-H2*. This relates to the fact that the pre-vernalisation flowering repressor *Vrn-H2* (Yan *et al.*, 2004) is deleted in spring barley cultivars such as Barke (von Zitzewitz *et al.*, 2005). In spring-sown field trials without exposure to severe cold such as that found in Dundee and Halle, the initiation of inflorescence is delayed under the presence of the functional *Hsp* allele of *Vrn-H2*. In addition, broad variations of family-specific *Hsp* effects were observed for SHO and HEA. This was particularly true for Dundee, where some *Hsp* alleles seem to have had no delaying effect on SHO and HEA whereas other *Hsp* alleles delayed them by 2.0 d (HEB families 01, 10, and 12) and 2.3 d (HEB family 03), respectively. Families with a strong delaying, effect especially for SHO in Dundee, seem to have originated predominantly from coastal areas of the ‘Fertile Crescent’ (Supplementary Fig. S5). Interestingly, *Vrn-H2* possibly exerted a pleiotropic effect on HEI in a location-specific manner. In Dundee, HEI was increased by up to 6.4 cm in HEB family 04, whereas in Halle it was reduced by up to 4.5 cm in HEB family 09. The effect of *Vrn-H2* on plant height was also found in a barley RIL population developed at ICARDA in Syria (Rollins *et al.*, 2013). In addition, the rice *VRN2*-like gene *Ghd7* was shown to exhibit pleiotropic effects on plant height and other yield-related traits (Xue *et al.* 2008; Greenup *et al.*, 2009). To date, very little is known about the direct or indirect molecular effects of flowering-time genes on plant architecture. The contrasting effects of *Hsp* in HEB families 04 and 09 may be a good starting point for a deeper study of the potential HEI effects caused by *Vrn-H2*.

### QTL region 6H-1

This unknown QTL is mainly characterised by strong family-specific and location-specific effects in the HEB-25 population. For example, the *Hsp* allele present in HEB family 15 increased HEI by 6.7 cm in Dundee (with no effect in Halle) and in HEB family 24 it increased SHO and HEA in Halle by 3.5 and 4.3 d, respectively (with no effect in Dundee). No candidate gene explaining these *Hsp* effects has so far been located in the QTL-6-1 region. We intend to fine-map and, ultimately, clone these family-specific and location-specific *Hsp* effects in follow-up studies. Segregating high-resolution sub-populations derived from informative HEB lines, which were genotyped as heterozygous in the respective QTL regions, are available and can be used as starting points for such studies.

### QTL region 7H-3 (*Vrn-H3*)

QTL region 7H-3 showed significant effects for most developmental traits at both locations except for HEI and RIP, for which effects were only detected at one location. A number of QTLs showed family-specific effects. For example, the *Hsp* alleles decreased HEA by 2.0 d and 1.5 d in HEB families 24 and 13, respectively, but increased HEA by 6.6 d in family 12. For most traits markers, SNP 12_30894 and SNP 12_30895, which are directly located in the *Vrn-H3* gene, showed the highest significance. *Vrn-H3* (also named *HvFT1*) is a homologue of the Arabidopsis *FLOWERING LOCUS T* gene (Yan *et al.*, 2006) and is the main integrator of photoperiod and vernalisation signals, which facilitate the switch from the vegetative to reproductive phase of the plant. The allelic diversity in terms of family-specific effects in the HEB-25 population corresponds to previous studies, which also found ample natural variation in the promoter and first intron of *HvFT1* (Loscos *et al.*, 2014). In addition, different promoter haplotypes of *HvFT1* have been identified and associated with late- and early-flowering phenotypes in a collection of barley landraces (Yan *et al.*, 2006; Casas *et al.*, 2011). Moreover, Loscos *et al.* (2014) and Nitcher *et al.* (2013) have pointed out that a broad range of copy number variation is present at the *HvFT1* locus. An increased copy number of the transcribed region (without promoter) was associated with an acceleration of flowering time. We assume that the HEA variation observed in HEB-25 noted above was also caused by sequence variation among the *Hsp* alleles of *HvFT1* (*Vrn-H3*). We are currently extending exome capture sequencing based on Mascher *et al.* (2013) to include all the HEB-25 lines in order to better explain the causes of the family-specific effects of *HvFT1*. This effort may help to relate sequence variation among *HvFT1* alleles to *Hsp* effects on HEA and other developmental traits in the HEB-25 population.

### Plant development and its impact on lodging and tiller thickness

Plant architectural characteristics, including tiller thickness (TCK) and resistance to lodging (LOD), are important traits for breeding. The heavy spikes of potentially high-yielding cultivars may lead to poor harvestable yields and grain quality if the LOD and TCK characteristics are unfavourable (Caierão, 2006; Dockter and Hansson, 2015). In Halle, we observed negative correlations between LOD and plant development traits (*r*_LOD×SHO_=–0.52, *r*_LOD×HEA_=–0.34, *r*_LOD×MAT_=–0.40) and a positive correlation with HEI (*r*_LOD×HEI_=0.65). The latter effect can be partially explained through pleiotropic effects of *sdw1* containing QTL 3H-11, where the *Hsp* allele increased HEI by 18.1 cm and LOD by a score of 1.1 in Halle. Likewise, the *Vrn-H2*-containing QTL 4H-8 also showed pleiotropic effects, with the *Hsp* allele decreasing HEI by 3.1 cm and LOD by a score of 0.2 in Halle. Overall, there was a high similarity between the LOD QTL described here in this study and that of Tondelli *et al.* (2013). For TCK, a further parameter used to characterise robustness of plant stems, reliable major QTLs were detected. The *Hsp* alleles of *Ppd-H1* (QTL 2H-2) and *HvCEN* (QTL 2H-3), both producing accelerating effects on plant development, showed negative effects on TCK, reducing it by scores of 1.4 and 1.6, respectively, in Dundee. We conclude that earliness of development seems to have a decisive negative impact on TCK. However, the overall effect of *Hsp* alleles at the *uzu* locus (QTL 3H-5) increased TCK by a score of 1.3 in Dundee without showing any crucial effect on plant development. We therefore conclude that a further, so far unknown, physiological process may also contribute to the expression of TCK in the HEB-25 population. Since lodging did not occur in Dundee, no direct correlation between LOD and TCK could be estimated. However, since only one QTL region (4H-1) showed corresponding QTLs for LOD and TCK, we assume that both traits are primarily controlled independently, at least under low to medium lodging pressure.

## Conclusions

The ultimate goal of GWAS is to identify gene alleles that control phenotypic diversity in breeding pools (Thomas *et al.*, 2011). In classical SNP-based bi-parental QTL approaches only two alleles can be distinguished. In our study we showed that cumulative significant SNP effects (Maurer *et al.*, 2017) can be applied to identify family-specific QTL effects of exotic alleles and to make use of the full potential of the multi-parental barley NAM population HEB-25. We have found several exotic alleles that showed reliable family-specific effects for all investigated traits, even with differing effect directions. This finding provides the ability to fine-tune traits for plant development including flowering time, an important co-determinant of yield (Jung and Müller, 2009). The selection of exotic alleles to fine-tune yield-related traits may ultimately result in cultivars that are better adapted to specific environments.

In the present study, the HEB-25 population was studied under two contrasting climatic environments, Halle (Germany) and Dundee (Scotland), to investigate location-specific QTL effects on plant development. The longer growing season in Dundee is presumably caused by lower temperatures and slightly higher rainfall and day length during the summer, resulting in a delayed plant development compared to Halle. This effect is reinforced in Halle through higher temperatures in July, which accelerate plant development and maturation in a long day-dependent manner (Hemming *et al.*, 2012). Several QTL regions seemed to interact significantly with the contrasting environments. These QTL ×environment interactions are essential for sessile plants to regulate physiological processes and to ensure that the optimal developmental stage occurs at the right seasonal time. This adaptation capacity may be valuable with regard to climate change, where higher temperatures and drought are predicted to reach Northern Europe (Lobell *et al.*, 2011; Olesen *et al.*, 2011). This raises the question as to how adaptable are cultivars with an insensitive *ppd-H1* allele, which is prevalent in Central and Northern European cultivars? Photoperiod-insensitive *ppd-H1* alleles have been favoured in regions with long growing seasons and good water availability, such as Central Europe, due to the ecological advantages they confer. In large parts of Southern Europe and south-west Asia, however, the sensitive *Ppd-H1* allele is predominant (Cockram *et al.*, 2007; Jones *et al.*, 2008). The current long growing season in Northern Europe might increasingly change towards Mediterranean conditions and the ecological advantages of *ppd-H1* could thus disappear in some regions. Our results suggest that the sensitive exotic *Ppd-H1* alleles not only perceive day length but also interact with temperature to regulate plant development in barley. In this regard, Borràs-Gelonch *et al.* (2011) and Ejaz and von Korff (2017) have also underlined the interaction of *Ppd-H1* and temperature in barley in different genetic backgrounds and environments. To overcome future agricultural challenges in a rapidly changing environment, the *Ppd-H1*-dependent adaptation potential, which requires a shortened plant cycle under dry summer cultivation, may gain more importance in Central Europe. The observed latitudinal cline of the *Ppd-H1* allele in Europe could thus move further north after sensitive alleles have been introduced, for example by using the *Hsp* alleles of the multi-parental HEB-25 population that have been characterised here.

## Supplementary data

Supplementary data are available at *JXB* online.

Fig. S1. Climate data for both locations during the experimental period.

Fig. S2. Frequency distribution of BLUEs for all traits, plotted as density histograms.

Fig. S3. Frequency distribution of BLUEs for SHO, HEA, and MAT as a function of day length.

Fig. S4. Family-specific effects of all the QTLs discussed.

Fig. S5. Geographic origin of HEB-25 donors with regard to QTL effects of the exotic *Vrn-H2* alleles on shooting.

Table S1. ANOVA results of phenotypic data within locations.

Table S2. GWAS output of all significant SNPs across all cross-validation (CV) runs.

Table S3. Family-specific effect estimation based on the cumulating method.

Table S4. List of QTLs, extreme family effects, and candidate genes calculated for eight traits.

Table S5. Presence of SNPs inside QTL candidate genes in HEB families based on exome capture sequencing.

Supplementary Figures

Supplementary Tables
