# Supplementary material for: Contrasting genetic regulation of plant development in wild barley grown in two European environments revealed by nested association mapping
Source: J Exp Bot. 2018 Jan 18;69(7):1517–31. doi: 10.1093/jxb/ery002 (PMC5888909; doi:10.1093/jxb/ery002)
Supplement: Supplementary Figures [file ery002_suppl_figures_s1-s5.pdf]

Figure S1. Climate diagrams of both locations during the experimental period.

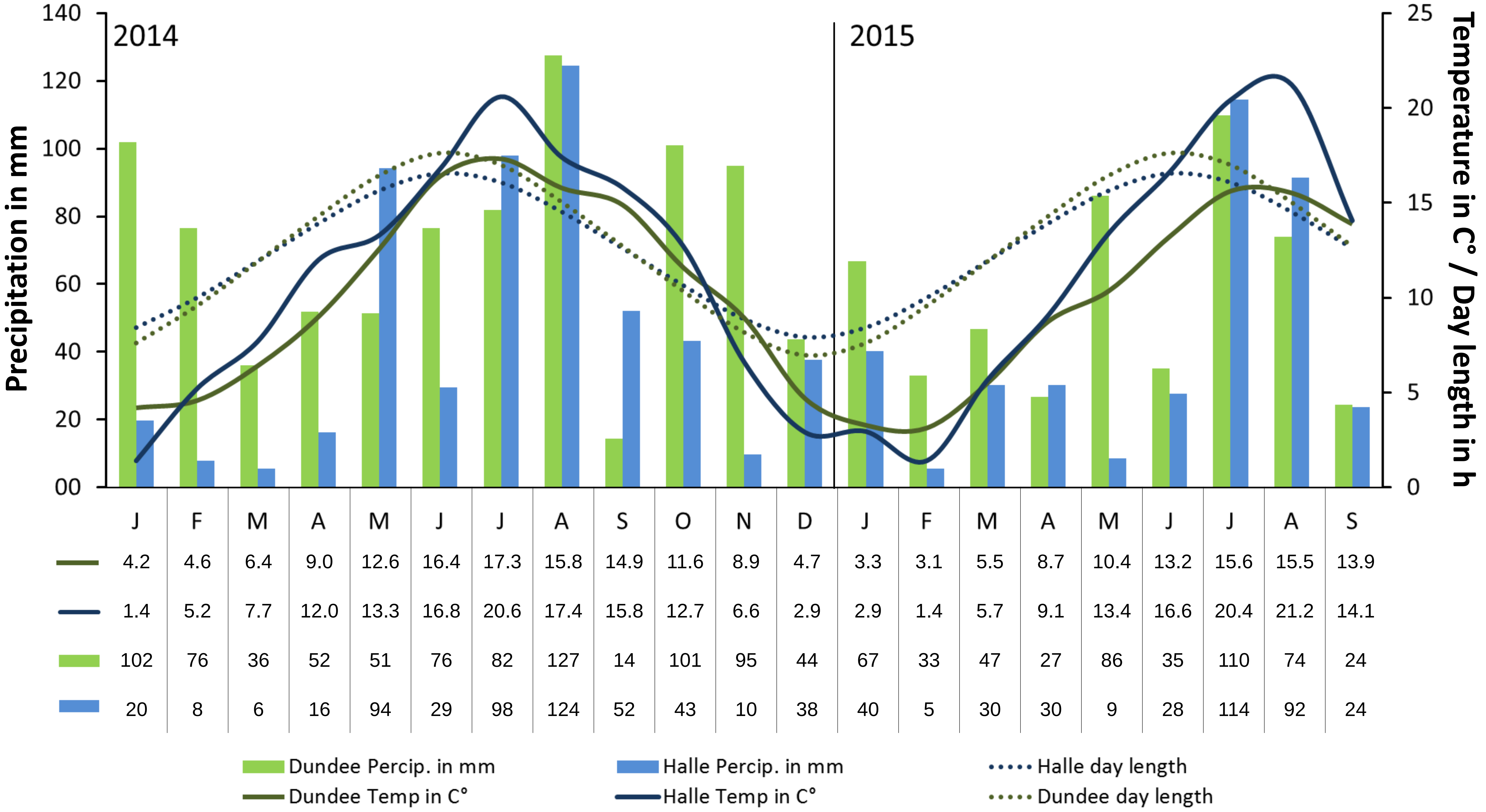

	J	F	M	A	M	J	J	A	S	O	N	D	J	F	M	A	M	J	J	A	S
Dundee Temp in C°	4.2	4.6	6.4	9.0	12.6	16.4	17.3	15.8	14.9	11.6	8.9	4.7	3.3	3.1	5.5	8.7	10.4	13.2	15.6	15.5	13.9
Halle Temp in C°	1.4	5.2	7.7	12.0	13.3	16.8	20.6	17.4	15.8	12.7	6.6	2.9	2.9	1.4	5.7	9.1	13.4	16.6	20.4	21.2	14.1
Dundee Percip. in mm	102	76	36	52	51	76	82	127	14	101	95	44	67	33	47	27	86	35	110	74	24
Halle Percip. in mm	20	8	6	16	94	29	98	124	52	43	10	38	40	5	30	30	9	28	114	92	24

Precipitation (bar chart), temperatures (solid lines) and day length (dotted lines) as monthly average of Halle (GER) and Dundee (UK) during 2014 and 2015.

Figure S2. Frequency distribution of BLUEs for all traits, plotted as density histograms.

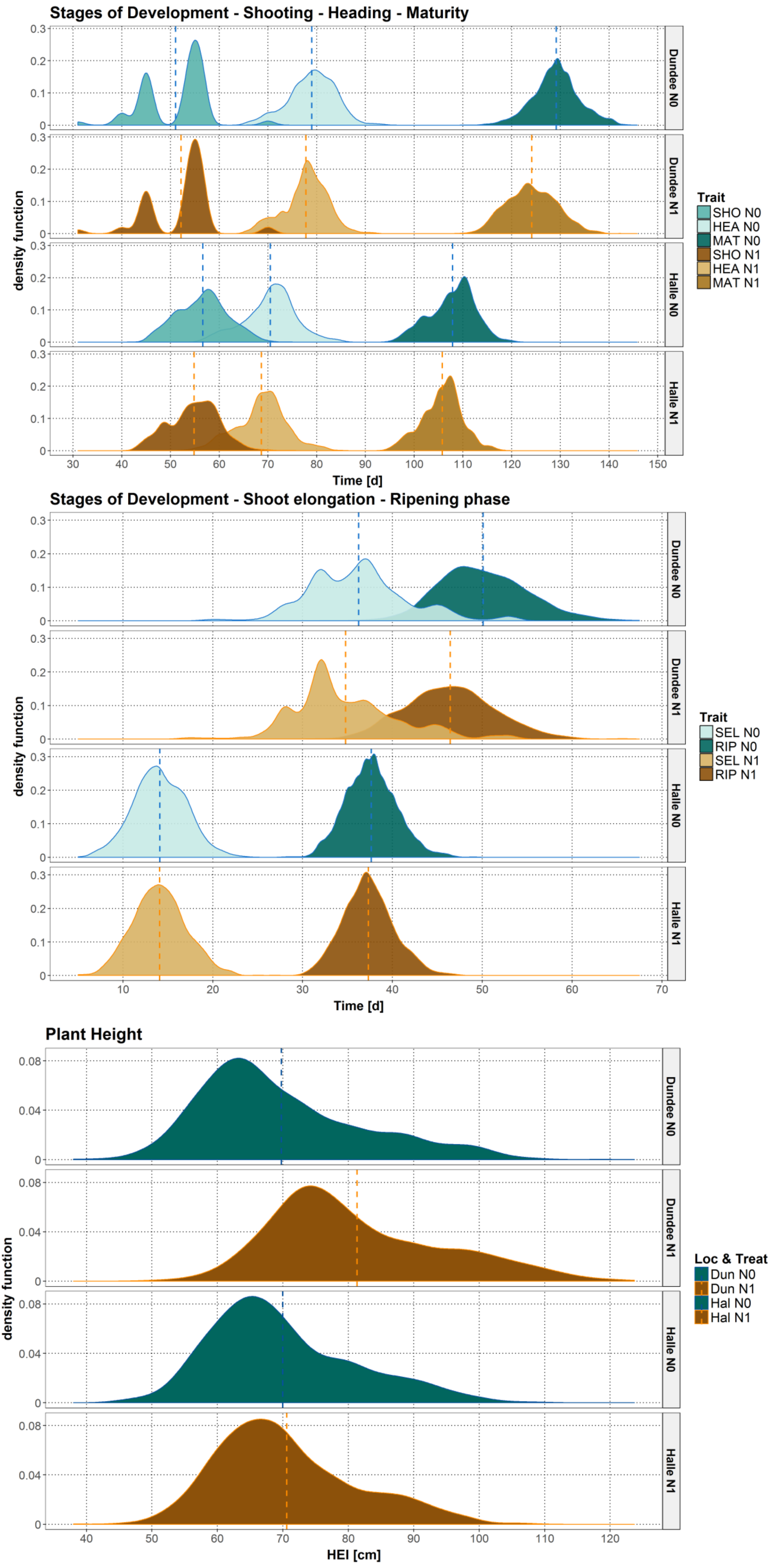

Figure S3. Frequency distribution of BLUEs for SHO, HEA and MAT as a function of day-length.

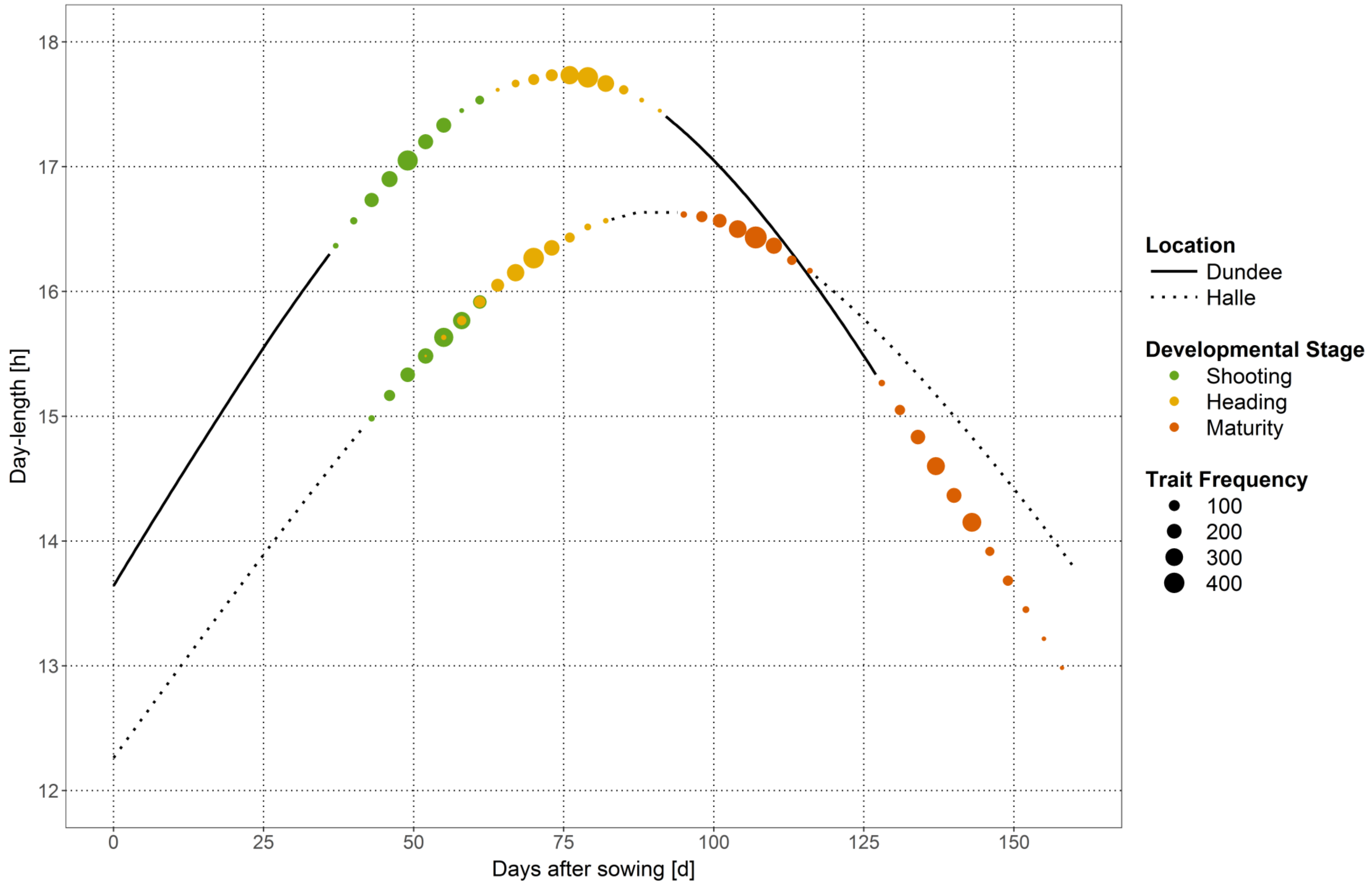

Mean day-length and plant development at each testing site. Data are BLUEs of two years and two treatments of the HEB-25.

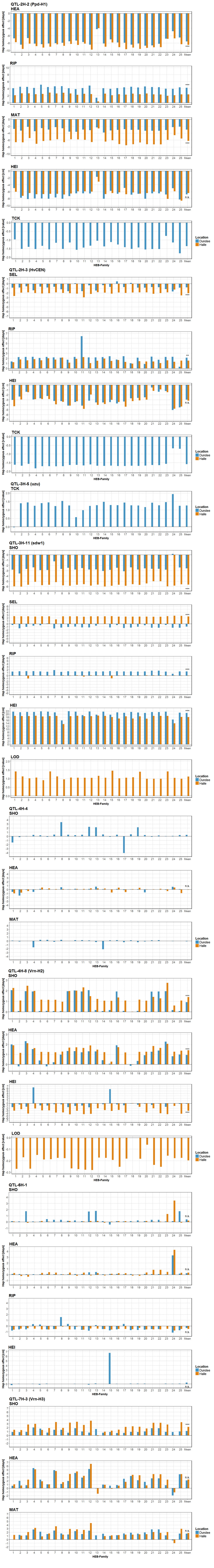

QTL-2H-2 (Ppd-H1)
HEA
RIP
MAT
HEI
TCK
QTL-2H-3 (HvCEN)
SEL
RIP
HEI
TCK
QTL-3H-5 (uzu)
TCK
QTL-3H-11 (sdw1)
SHO
SEL
RIP
HEI
LOD
QTL-4H-4
SHO
HEA
MAT
QTL-4H-8 (Vrn-H2)
SHO
HEA
HEI
LOD
QTL-6H-1
SHO
HEA
RIP
HEI
QTL-7H-3 (Vrn-H3)
SHO
HEA
MAT
Hap homozygous effect [days]
Hap homozygous effect [cm]
Hap homozygous effect [value]
HEB-Family
Mean
Location
Dundee
Halle
***
n.s.

Figure S5. Origin of HEB-25 donors in regard to QTL effects of exotic *Vrn-H2* alleles on shooting.

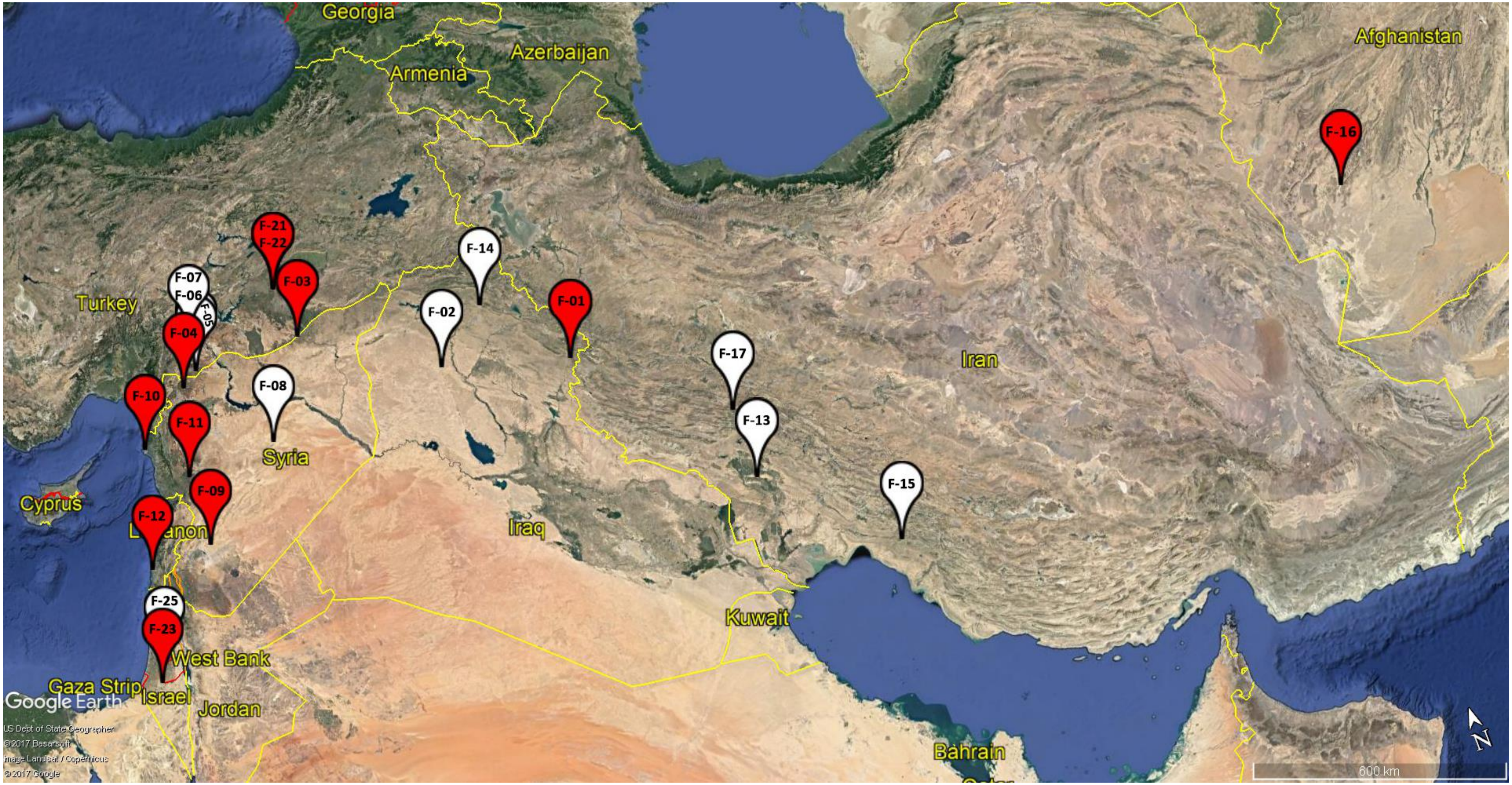

HEB families with a strong delaying effect (> 1.9 days) of the exotic *Vrn-H2* allele on SHO, especially in Dundee
HEB families with a low delaying effect (<0.2 days) of the exotic *Vrn-H2* allele on SHO, especially in Dundee

Origin of the HEB donors in the Fertile Crescent (if known). F-24, the only *Hordeum vulgare* ssp. *agriocrithon* accession originating from Tibet, China. Exotic *Vrn-H2* alleles with a strong delaying effect predominantly originate from coastal areas.
